# Role of FXR Agonist in Cholestasis Following Live Donor Liver Transplantation: A Randomized Open-Label Trial

**DOI:** 10.7759/cureus.97915

**Published:** 2025-11-27

**Authors:** Anila KN, Saraswathy S, Unnikrishnan G, Dinesh Balakrishnan, Shweta Mallick, Nafiya Muhammed Zackariah, Haritha Rajakrishnan, Rajana Gopinath, Sajitha Krishnan, Sudhindran S

**Affiliations:** 1 Department of Gastrointestinal Surgery and Solid Organ Transplantation, Amrita Institute of Medical Sciences and Research Centre, Amrita Vishwa Vidyapeetham, Kochi, IND; 2 Department of Biochemistry, Amrita Institute of Medical Sciences and Research Centre, Amrita Vishwa Vidyapeetham, Kochi, IND

**Keywords:** alkaline phosphatase, cholestasis, fxr agonist, live donor liver transplantation, obeticholic acid, ursodeoxycholic acid

## Abstract

Background: Intrahepatic cholestasis is a frequent postoperative issue in living donor liver transplantation (LDLT), typically stemming from causes such as reperfusion-related damage, infections, biliovascular issues, or rejection. Prompt intervention is key to preserving graft viability and promoting long-term recovery.

Objective: To evaluate the safety and efficacy of obeticholic acid (OCA), a farnesoid X receptor (FXR) agonist, versus ursodeoxycholic acid (UDCA) in post-liver transplant patients to ameliorate cholestatic injury and reduce biopsy-proven rejection.

Methodology: In this randomized, open-label trial, adult LDLT recipients received OCA (5 mg/day) or UDCA (300 mg thrice daily) from postoperative day 3 for one year. The primary endpoint was ≥15% reduction in alkaline phosphatase (ALP) and gamma-glutamyl transferase (GGT) from one-month postoperative baseline assessed at three, six, and 12 months. Secondary outcomes included changes in relevant biochemical tests and molecular parameters like plasma bile acids, transforming growth factor-beta (TGF-β), cytokeratin-18 (CK-18), serum autotaxin, fibroblast growth factor-19 (FGF-19), and bile salt export pump (BSEP), as well as the incidence of rejection, early allograft dysfunction (EAD), biliary complications, mortality, adverse events, and quality of life (QoL).

Results: In the interim analysis of 83 patients, more than a 15% reduction in ALP between six and 12 months was observed in a higher proportion of patients in the OCA group (43%) than in the UDCA group (21%) (p=0.036). More than a 15% reduction in GGT between one and three months was observed in 80% of patients in the OCA group compared to 62% in the UDCA group (p=0.062). Plasma bile acids were lower in the OCA group at one month (170 vs. 226 µmol/L; p=0.012). Low-density lipoprotein (LDL)-cholesterol was higher in the OCA group at three (OCA 121 vs UDCA 107, p=0.027) and six months (OCA 130 vs UDCA 117, p=0.043). UDCA therapy resulted in a significantly greater improvement in overall QoL, with higher gains in both total mean and domain scores compared to OCA. No differences were detected in rejection, biliary complications, mortality, or other secondary endpoints.

Conclusion: OCA led to greater biochemical improvement, while better QoL was achieved with UDCA in LDLT recipients, with a comparable safety profile.

## Introduction

Liver disease poses a significant global health challenge, accounting for approximately two million deaths annually and representing around 4% of all deaths worldwide [1, 2]. Liver transplantation (LT) is the only curative option for individuals with decompensated chronic end-stage liver disease or acute liver failure (ALF) [3]. Living-donor LT (LDLT) has become increasingly important in liver transplant programs worldwide, particularly in regions with limited access to deceased-donor organs. Approximately 8,000 LDLTs are performed each year globally, constituting nearly one-quarter of all liver transplants [4, 5].

One of the major challenges following transplantation, especially LDLT, is intrahepatic cholestasis, which significantly contributes to post-transplant morbidity [6]. Several mechanisms can lead to the development of cholestasis in this context. A primary cause is ischemia-reperfusion injury, which occurs due to a period of reduced blood supply during organ procurement, followed by reoxygenation after implantation. This process induces oxidative stress and cellular injury, often manifesting as cholestasis in the postoperative phase [7]. Infections, whether bacterial, viral, or fungal, are also common after transplantation and can exacerbate cholestasis through the production of pro-inflammatory cytokines and subsequent hepatocellular injury.

Additionally, drug-induced liver injury from commonly used post-transplant medications, such as immunosuppressants, antimicrobials, and antifungals, can disrupt bile synthesis and transport, leading to cholestatic patterns of injury [7]. Immune responses against the transplanted liver, such as T-cell-mediated rejection, antibody-mediated rejection, or immune-mediated cholangitis involving CD8⁺ lymphocytes, can impair bile flow and damage bile ducts. Technical complications during surgery, including issues at the biliary or vascular anastomosis, may also cause narrowing or obstruction, resulting in both intrahepatic and extrahepatic cholestasis. Notably, the pathophysiological features of post-transplant cholestatic complications share similarities with chronic cholestatic liver conditions such as primary biliary cholangitis (PBC) and metabolic-associated steatohepatitis (MASH) [8, 9].

Effective pharmacological treatment for cholestasis remains elusive, with few agents demonstrating consistent benefit. Ursodeoxycholic acid (UDCA) is routinely employed in liver transplant recipients for its hepatoprotective properties, often initiated at 10-15 mg/kg twice daily for approximately three months. However, its effectiveness in managing post-transplant cholestasis remains debated, with clinical outcomes showing inconsistent results [10]. Obeticholic acid (OCA), a synthetic derivative of bile acids and a potent, selective farnesoid X receptor (FXR) agonist, received U.S. Food and Drug Administration (FDA) approval in 2016 for use alongside UDCA in the treatment of PBC [11]. Activating the FXR receptor is crucial for various liver physiological functions, particularly in regulating bile acid concentrations within hepatocytes. OCA enhances bile homeostasis through two primary mechanisms: reducing bile production and increasing bile excretion from hepatocytes into the biliary canaliculi [12, 13]. With 100 times the potency of chenodeoxycholic acid, OCA has shown superior efficacy over UDCA in treating PBC and primary sclerosing cholangitis (PSC). However, the effectiveness of OCA in recipients of LDLT remains inadequately studied. Our study aims to assess the efficacy of OCA compared with UDCA in alleviating cholestatic injury in recipients of LDLT.

## Materials and methods

Study design and participants

This parallel-group, open-label, randomized controlled trial was conducted in patients who underwent LDLT between December 2021 and July 2023 at the Department of Gastrointestinal Surgery at Amrita Institute of Medical Science, a tertiary care academic hospital in Kochi, India.

Eligibility and exclusion criteria

Inclusion Criteria

Adult recipients (≥18 years) of LDLT during the study period were included.

Exclusion Criteria

Recipients younger than 18 years, patients who underwent deceased-donor LT, transplants performed for ALF, and patients who did not survive beyond 30 days post transplant were excluded from the study. Written informed consent was obtained prior to surgery during study enrolment.

Study endpoints

The primary composite endpoint was a >15% reduction in alkaline phosphatase (ALP) or gamma-glutamyl transferase (GGT) levels from baseline (one month post LT) to the third, sixth, and 12^th^ months.

Secondary Endpoints

Biochemical and molecular indices between the two study groups: Absolute values of ALP, GGT, total bilirubin, direct bilirubin, aspartate aminotransferase (AST), alanine aminotransferase (ALT), prothrombin time (PT)/international normalized ratio (INR), total proteins, globulins, albumin, and platelets; Lipid profile: total cholesterol, low-density lipoprotein (LDL), high-density lipoprotein (HDL), very LDL (VLDL), triglycerides (TG), glycosylated haemoglobin (HbA1c), fasting blood sugar (FBS), C-reactive protein (CRP). The level of fibroblast growth factor-19 (FGF-19) is used to assess FXR activation. FGF-19 is an enterokinase released after FXR activation that binds to FGF-4 receptor and suppresses transcription of cholesterol 7 alpha-hydroxylase (CYP7A1), the key enzyme for converting cholesterol to bile acids in hepatocytes [14,15]. Plasma bile acid levels, a sensitive indicator for detecting mild or early cholestasis [16]; Serum autotaxin, an enzyme that converts lysophosphatidyl choline to lysophosphatidic acid, a signalling molecule for cell proliferation, inflammation, and fibrosis; levels correlate with cholestatic pruritus [17]; Cytokeratin 18 (CK-18), expressed in hepatocytes and biliary epithelium; fragments are released into the bloodstream during necrosis or apoptosis, reflecting the extent of apoptosis and fibrosis [18]; Transforming growth factor β (TGF-β), which stimulates hepatic stellate cell activation, driving extracellular matrix deposition and fibrosis; also acts as an immunomodulator that may contribute to rejection [19]; Bile salt export pump (BSEP) levels, a bile acid transporter on the canalicular membrane of hepatocytes, pump bile acids into bile and are normally not present in circulation; they are detectable in certain conditions such as drug-induced cholestasis and measurable using sandwich enzyme-linked immunosorbent assay (ELISA) [20, 21].

Other Secondary Outcomes

Incidence of biliary complications: Rate of acute cellular rejection (biopsy-proven and treated biochemical rejection). Biopsy-proven acute cellular rejection: an episode of T cell-mediated rejection confirmed by biopsy and managed with corticosteroids or lymphocyte-depleting agents; diagnosis based on Rejection Activity Index score and Banff criteria [22]. Treated biochemical rejection: threefold rise in transaminases without evident biliovascular or infective cause, resolving with pulsed steroids; biopsy not performed due to contraindications (coagulopathy or ascites), early allograft dysfunction (EAD) [23]; Adverse drug reactions (assessed using the Naranjo scale); All-cause mortality rate; Health-related quality of life (QoL) (measured using the Chronic Liver Disease Questionnaire (CLDQ))

Recruitment and randomisation

Participants were randomized by a clinical pharmacist using a permuted block sequence of computer-generated random numbers into either the standard arm (UDCA-URSETOR®, Torrent Pharmaceuticals, Ahmedabad, India) or the study arm (OCA-OCANASH®, Macleods Pharmaceuticals, Mumbai, India). The sequence was kept in sealed, opaque envelopes and opened by the pharmacist on the third day following LDLT. The standard arm received UDCA 300 mg thrice daily, while the study arm received OCA 5 mg once daily, both for one year.

Post-transplant management

The immunosuppressive protocol comprised corticosteroids, tacrolimus, and mycophenolate mofetil. In patients with current or prior renal impairment, induction immunosuppression with basiliximab (an interleukin-2 receptor antagonist) was used. Tacrolimus was initiated at 0.5 mg twice daily, titrated to maintain a target trough of 5-7 ng/mL. Mycophenolate mofetil was started at 500 mg twice daily, unless contraindicated by a platelet count < 50 × 10⁹/L or a total leukocyte count < 4 × 10⁹/L. Steroids were started at 30 mg/day and tapered over two months.

Data collection

In-hospital data, including organ dysfunction, rejection episodes, hospital stay, and mortality, were recorded prospectively. Follow-up visits were scheduled at one week, one month, three months, six months, and 12 months post transplant for physical examination and safety assessments.

Any biliary strictures, leaks, or anastomotic complications were documented. EAD was evaluated using Olthoff et al. criteria [23].

Adverse drug reactions were recorded in the case report form, and causality was assessed using the Naranjo scale (classified as definite, probable, possible, or unlikely) [24].

QoL was measured using the validated CLDQ pre-transplant and at one year post transplant. Copyright for the English version was obtained from the authors. The questionnaire was administered in either Malayalam or English, based on participant preference (Appendix A). It assessed six domains: Abdominal Symptoms (AB), Fatigue (FA), Systemic Symptoms (SY), Activity (AC), Emotional Function (EM), and Worry (WO). Scores ranged from 0 to 203, with lower scores indicating poorer QoL [25].

Secondary endpoint molecular markers, including total bile acids, FGF-19, TGF-β, BSEP, CK-18, and serum autotaxin, were assessed using an ELISA (ELK Biotechnology™, ELK (Wuhan) Biotechnology Co., Ltd., Wuhan, China) at the centralized biochemistry laboratory. Blood samples collected during the one-month and twelfth-month post-transplant follow-up visits are being used for ELISA testing.

Sample size

The sample size was based on a pilot study comparing OCA and UDCA in post liver transplant patients. A difference in ALP levels was observed (5.131 ± 39.639 for OCA vs. 12.496 ± 52.139 for UDCA). Assuming 80% power and a 95% confidence level, the minimum required sample size was calculated as 108 patients per group. Although the calculated sample size was 108 per group, this interim analysis includes data from approximately 41 patients per group who had completed the scheduled follow-up at the time of interim assessment.

Statistical analysis

Categorical Variables

presented as frequency and percentage; compared using Pearson’s chi-squared test or Fisher’s exact test. Normally distributed continuous variables: presented as mean ± standard deviation (SD); compared using an independent sample t-test. Non-normally distributed continuous variables: compared using the Mann-Whitney U test. Within-group changes (baseline vs. three, six, and 12 months): analyzed using paired t-tests or Wilcoxon signed-rank tests. Pre- and post-treatment quality of life scores were compared using the Wilcoxon signed-rank test. Differences in median change scores of CLDQ domains and total score between OCA and UDCA groups were assessed with the Mann-Whitney U test. Statistical analysis was performed using IBM SPSS Statistics software, version 20.0 (IBM Corp., Armonk, NY).

This report presents an interim intention-to-treat (ITT) analysis including all participants who had completed the scheduled one-year follow-up at the time of analysis. Of the 172 randomized participants, 83 had completed one year of follow-up and were included in this interim analysis. Patients who had not yet completed their scheduled one-year follow-up were not included in the current analysis. All enrolled participants were contacted in person during outpatient visits or via telephone for follow-up if appointments were missed. As there were no missing data among those included, no imputation methods were required. The final analysis, which will include all randomized participants, is planned at study completion and will provide a more definitive assessment of treatment efficacy and safety.

Ethical approval

Informed consent was obtained from all subjects involved in the study. The trial was carried out in accordance with the Declaration of Helsinki and adhered to established good clinical practice guidelines. This study is approved by the Institutional Ethics Committee, Amrita Institute of Medical Science, Kochi (approval number: IEC-AIMS-2021-GISURG-262). The study has also obtained Clinical Trial Registry of India (CTRI) registration (CTRI/2021/11/038218).

## Results

Participant flow and baseline

**Figure 1:**
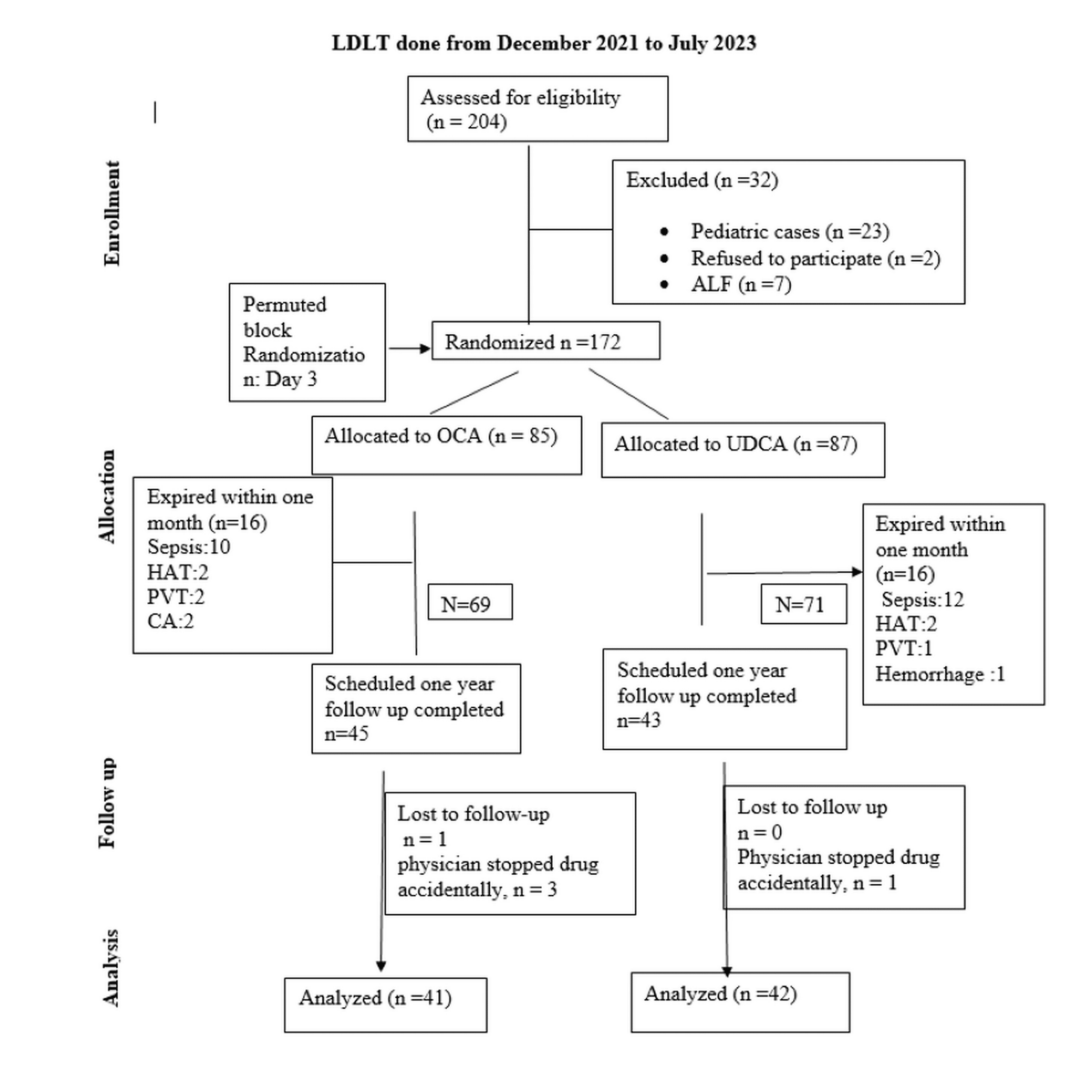
CONSORT diagram of the interim analysis CONSORT: Consolidated Standards of Reporting Trials; LDLT: living donor liver transplantation; ALF: acute liver failure; OCA: obeticholic acid; UDCA: ursodeoxycholic acid; HAT: hepatic artery thrombosis; PVT: portal vein thrombosis

During this interim analysis, 204 patients were assessed for eligibility, of whom 32 were excluded in the initial phase due to pediatric status or urgent transplantation for ALF. The remaining 172 patients were randomly assigned to the OCA or UDCA groups. Among the 83 patients included in the present analysis, the median age was 52 years (IQR 45-57). There was no missing data among participants who completed the scheduled follow-up. One patient in the OCA group was lost to follow-up (Figure 1). Therefore, this interim analysis was conducted on participants who had complete follow-up data and remained on their assigned treatment.

Baseline characteristics, which were comparable between both groups of recipients, are outlined in Table 1. Alcohol-induced liver disease was the predominant etiology for transplantation, with a prevalence of 60%(n=25) in the OCA group and 68% (n=28) in the UDCA group. The second most common cause was metabolic dysfunction-associated steatotic liver disease (MASLD) (OCA 31% (n=13) vs. UDCA 29% (n=12)). Among the transplanted patients, acute-on-chronic liver failure (ACLF), as defined by the European Association for the Study of the Liver (EASL) criteria [3], was present in 22% (n=9) of the UDCA group and 19% (n=8) of the OCA group, while the remaining patients had decompensated chronic liver disease (DCLD). Donor characteristics and relevant preoperative graft parameters are also presented in Table 1 below.

**Table 1:** Demographic details, donor characteristics and pre-postoperative data ^1^Median (Q1, Q3); n (%); ^2^Wilcoxon rank sum test; Fisher's exact test; Pearson's Chi-square test *OCA: obeticholic acid; UDCA: ursodeoxycholic acid; AKI: acute kidney injury; ACLF: acute-on-chronic liver failure; MASLD:  metabolic-associated steatotic liver disease

Characteristic	Overall, N = 83^1^	OCA, N = 42^1^	UDCA, N=41^1^	p-value^2^
Recipient parameters: Age (years)	52 (45, 57)	50 (45, 57)	54 (45, 58)	0.4
Gender: Males	79 (95%)	40 (95%)	39 (95%)	0.9
Weight (kg)	74 (66, 81)	74 (66, 82)	74 (68, 80)	0.9
Comorbidities				
Diabetes	32 (39%)	16 (38%)	16 (39%)	0.9
Hypertension	13 (16%)	5 (12%)	8 (20%)	0.3
Dyslipidaemia	2 (2.4%)	0 (0%)	2 (4.9%)	0.2
AKI*	1 (1.2%)	1 (2.4%)	0 (0%)	0.9
Model for End-stage Liver Disease Score (MELD)	24.0 (20.0, 28.0)	23.5 (21.0, 28.0)	24.0 (19.0, 28.0)	0.7
Alternative medicine therapy	29 (35%)	15 (36%)	14 (34%)	0.9
Indication for Transplantation:				
Decompensation	60 (72%)	30 (71%)	30 (73%)	0.9
ACLF*	17 (20%)	8 (19%)	9 (22%)	0.7
Hepatocellular carcinoma	6 (7%)	4 (10%)	2 (5%)	0.7
Etiology				0.7
Alcohol	53 (64%)	25 (60%)	28 (68%)	
MASLD*	25 (30%)	13 (31%)	12 (29%)	
Hepatitis B virus	1 (1.20%)	1 (2.44%)	0 (0.00%)	
Autoimmune	4 (4.82%)	2 (4.88%)	2 (4.76%)	
Wilson	1 (1.20%)	1 (2.44%)	0	
Donor parameters: Age (years)	38 (30, 45)	40 (32, 46)	37 (29, 45)	0.5
Gender: Female	53 (64%)	26 (62%)	27 (66%)	0.7
Weight	74 (65, 82)	75 (66, 83)	72 (64, 79)	0.6
Graft Parameters: Liver Spleen Attenuation Index	6 (1, 11)	7 (1, 12)	6 (-1, 11)	0.7
Liver/Spleen Index	1.10 (1.00, 1.23)	1.10 (1.01, 1.21)	1.10 (1.00, 1.23)	0.6
Graft weight (g)	729 (604, 800)	734 (640, 829)	718 (602, 792)	0.9
Graft-to-recipient weight ratio	1.04 (0.86, 1.21)	1.03 (0.89, 1.21)	1.05 (0.83, 1.20)	0.9
Warm Ischaemic time (min)	18 (13, 24)	18 (13, 23)	18 (13, 25)	0.9
Cold ischemic time (minutes)	56 (45, 67)	56 (45, 68)	56 (45, 63)	0.8
Immunosuppressants Used:				
Basiliximab	20 (24%)	8 (20%)	12 (29%)	0.47
Prednisolone	73 (88%)	36 (88%)	37 (88%)	>0.9
Tacrolimus	81 (98%)	40 (98%)	41 (98%)	>0.9
Mycophenolate	79 (95%)	41 (100%)	38 (90%)	0.12
Azoran	4 (4.9%)	0 (0%)	4 (9.8%)	0.12
Everolimus	36 (46%)	17 (43%)	19 (49%)	0.74

Immunosuppression

Basiliximab was used as induction immunosuppression in 20% of OCA patients and 29% of UDCA patients (χ²=0.5015, p=0.47). Tacrolimus, methylprednisolone, and mycophenolate mofetil were the main post-transplant immunosuppressive agents. In addition to tacrolimus, everolimus was used in 43% of patients in the OCA arm and 49% in the UDCA arm (χ²=0.1082, p=0.74).

Primary endpoint

Primary endpoint results are shown in Table 2. A Pearson’s chi-squared test revealed a statistically significant difference in the proportion of patients achieving >15% reduction in ALP between six and 12 months (OCA 43% (17) vs. UDCA 21% (8); χ²=4.413, p = 0.036). More than a 15% reduction in GGT between one and three months was observed in 80% of patients in the OCA group compared to 62% in the UDCA group (χ² = 3.485, p = 0.062). 

**Table 2:** Number of patients with 15% reduction in ALP and GGT at various time points - primary endpoints *OCA: obeticholic acid; UDCA: ursodeoxycholic acid; ALP: alkaline phosphatase; GGT: gamma glutamyl transferase

Characteristic	Overall, N = 83^1^	OCA, N = 41^1^	UDCA, N = 42^1^	p-value^2^
ALP				
1 to 3	36 (43%)	19 (46%)	17 (40%)	0.6
3 to 6	28 (34%)	13 (32%)	15 (36%)	0.7
1 to 6	29(35%)	18(44%)	11(26%)	0.091
6 to 12	25 (32%)	17 (43%)	8 (21%)	0.036
1 to 12	31(39%)	18(45%)	13(33%)	0.3
GGT				
1 to 3	59 (71%)	33 (80%)	26 (62%)	0.062
3 to 6	50 (60%)	27 (66%)	23 (55%)	0.3
1 to 6	62 (75%)	33(80%)	29 (69%)	0.2
1 to 12	60 (77%)	35 (88%)	25(66%)	0.023
6 to 12	25 (32%)	15 (38%)	10 (26%)	0.3

_^1^n (%)_				
_^2^Pearson's Chi-squared test_				

Absolute value assessment of clinical markers

Table 3 shows the absolute values of other liver function tests (total bilirubin, direct bilirubin, AST, ALT, total proteins, albumin, globulin, CRP, PT/INR, platelets, and tacrolimus levels) at different time points. Most results were comparable between the groups and did not reach statistical significance. The levels of immunosuppression did not differ significantly between the groups. Mean tacrolimus levels were 4.52 ± 1.94 in the OCA group vs. 3.96 ± 1.82 in the UDCA group at one month (W=1097; p=0.32). 

**Table 3:** Absolute value assessment of clinical markers ^1^ Median (Q1, Q3); ^2^ Wilcoxon rank sum test; Wilcoxon rank sum exact test *OCA: obeticholic acid; UDCA: ursodeoxycholic acid; TX: transplant; ALP: alkaline phosphatase; GGT: gamma glutamyl transferase; AST: aspartate aminotransferase; ALT: alanine aminotransferase; PT: prothrombin time; INR: international normalized ratio; CRP: C reactive protein; PLT: platelets

Characteristics by Timepoint	Overall, N = 83^1^	OCA, N = 41^1^	UDCA, N = 42^1^	p-value^2^
Post TX 1 week				
ALP	42 (34, 58)	43 (34, 58)	41 (34, 63)	0.8
GGT	117 (71, 221)	154 (88, 270)	94 (62, 157)	0.017
Total bilirubin	6.9 (5.4, 8.9)	4.3 (5.2, 8.7)	6.8 (5.6, 8.9)	0.9
Direct bilirubin	3.03 (2.16, 4.16)	3.02 (2.12, 4.03)	3.08 (2.29, 4.16)	0.6
AST	349 (205,636)	312 (179,714)	372 (223, 573)	0.6
ALT	289 (170, 514)	259 (156, 549)	342 (182, 510)	0.4
Albumin	3.10 (2.80, 3.60)	3.00 (2.80, 3.50)	3.20 (2.80, 3.70)	0.2
Globulin	1.45 (1.13, 1.97)	1.39 (1.15, 1.78)	1.46 (1.13, 2.15)	0.3
PT	35 (31, 42)	34 (29, 40)	36 (31, 43)	0.2
INR	2.83 (2.42, 3.44)	2.70 (2.43, 3.20)	2.89 (2.42, 3.60)	0.12
Total protein	4.70 (4.10, 5.20)	4.60 (4.10, 5.00)	4.80 (4.20, 5.40)	0.4
CRP	14(3, 26)	15(2, 28)	13(4, 23)	0.3
Platelet	57 (33, 81)	56 (40, 72)	59 (30, 88)	0.2
Peak values (1 week)				
Total bilirubin	4.14 (2.75, 6.20)	3.46 (2.62, 4.91)	4.62 (2.91, 6.41)	0.2
AST	272 (138, 469)	199 (129,415)	310 (161, 469)	0.14
ALT	333 (169, 577)	304 (167,505)	366 (187,642)	0.5
Month 1				
ALP	137 (97, 214)	147 (97, 273)	129 (97, 185)	0.2
GGT	97 (52, 261)	150 (70, 268)	90 (48, 190)	0.13
Total bilirubin	0.99 (0.64, 1.75)	0.99 (0.64, 1.75)	1.10 (0.64, 1.74)	0.8
Direct bilirubin	0.57 (0.32, 0.97)	0.59 (0.30, 1.22)	0.56 (0.36, 0.93)	0.8
AST	26 (15, 45)	24 (16, 46)	27 (14, 44)	0.9
ALT	33 (19, 68)	28 (19, 63)	40 (17, 68)	0.8
Albumin	3.60 (3.10, 4.00)	3.50 (3.00, 3.90)	3.60 (3.20, 4.00)	0.2
Globulin	2.64 (2.37, 3.17)	2.60 (2.39, 3.13)	2.64 (2.37, 3.23)	0.7
PT	13.80 (12.70, 15.20)	13.80 (12.80, 15.50)	13.85 (12.70, 14.90)	0.7
INR	0.98 (0.90, 1.11)	0.97 (0.91, 1.10)	1.00 (0.90, 1.12)	0.7
Total protein	6.40 (5.80, 6.80)	6.30 (5.90, 6.70)	6.50 (5.80, 6.90)	0.3
CRP	11 (4.35)	15 (4.59)	10 (3, 26)	0.2
PLT	161 (135, 233)	172 (143, 243)	158 (120,222)	0.2
Month 3				
ALP	132 (92, 166)	133 (93, 166)	132 (91, 162)	0.8
GGT	63 (33, 138)	63 (28, 161)	63 (34, 120)	0.7
Total bilirubin	0.71 (0.53, 1.05)	0.71 (0.50, 1.04)	0.76 (0.58, 1.05)	0.8
Direct bilirubin	0.33 (0.22, 0.49)	0.28 (0.19, 0.51)	0.37 (0.22, 0.46)	0.5
AST	25 (18, 33)	25 (18, 30)	25 (20, 37)	0.6
ALT	22 (15, 37)	20 (17, 33)	28 (15, 43)	0.2
Albumin	4.00 (3.60, 4.40)	4.00 (3.60, 4.30)	4.10 (3.70, 4.40)	0.5
Globulin	2.97 (2.67, 3.48)	3.19 (2.72, 3.55)	2.96 (2.58, 3.35)	0.3
PT	13.80 (12.90, 14.70)	13.50 (12.80, 14.40)	13.80 (13.00, 14.70)	0.3
INR	0.98 (0.91, 1.06)	0.96 (0.91, 1.03)	0.99 (0.91, 1.08)	0.2
Total protein	7.10 (6.50, 7.50)	7.10 (6.50, 7.50)	7.05 (6.60, 7.50)	>0.9
CRP	6 (3.14)	9 (3, 21)	6 (3,11)	0.4
PLT	161 (123, 205)	164 (121, 209)	152 (127,197)	0.6
Month 6				
ALP	126 (91, 196)	126 (91, 188)	127 (98, 205)	0.7
GGT	40 (21, 117)	53 (22, 127)	35 (21, 116)	0.7
Total bilirubin	0.86 (0.51, 1.22)	0.79 (0.52, 1.05)	0.87 (0.50, 1.36)	0.6
Direct bilirubin	0.29 (0.19, 0.55)	0.26 (0.20, 0.42)	0.33 (0.19, 0.58)	0.2
AST	25 (20, 39)	24 (19, 37)	26 (21, 46)	0.3
ALT	26 (14, 43)	25 (16, 35)	29 (14, 46)	0.3
Albumin	4.20 (3.70, 4.50)	4.10 (3.50, 4.40)	4.20 (3.70, 4.60)	0.2
Globulin	3.03 (2.65, 3.53)	3.14 (2.79, 3.57)	2.98 (2.56, 3.52)	0.2
PT	13.70 (12.90, 14.60)	13.80 (13.10, 14.30)	13.60 (12.70, 14.90)	0.3
INR	0.98 (0.91, 1.05)	0.98 (0.93, 1.02)	0.96 (0.90, 1.07)	0.3
Total protein	7.20 (6.90, 7.70)	7.30 (7.00, 7.50)	7.20 (6.80, 7.90)	>0.9
CRP	4 (2, 13)	5 (2, 15)	4 (2, 10)	0.5
PLT	144 (110, 199)	167 (119, 206)	140 (105, 179)	0.11
Month 12				
ALP	127 (86, 193)	123 (86, 203)	129 (89, 192)	0.6
GGT	37 (25, 114)	37 (26, 109)	41 (24, 129)	0.7
Total bilirubin	0.81 (0.58, 1.09)	0.81 (0.56, 1.31)	0.81 (0.60, 1.02)	0.6
Direct bilirubin	0.28 (0.21, 0.44)	0.27 (0.21, 0.45)	0.30 (0.21, 0.42)	0.8
AST	26 (20, 35)	26 (19, 35)	26 (21, 35)	0.7
ALT	23 (17, 41)	23 (15, 44)	24 (18, 35)	0.5
Albumin	4.30 (4.00, 4.50)	4.30 (3.75, 4.40)	4.40 (4.00, 4.60)	0.3
Globulin	3.22 (2.78, 3.51)	3.23 (2.72, 3.54)	3.10 (2.79, 3.50)	0.7
PT	13.85 (12.90, 14.90)	13.65 (12.65, 15.05)	13.95 (13.40, 14.90)	0.7
INR	0.96 (0.89, 1.04)	0.95 (0.89, 1.06)	0.97 (0.92, 1.03)	0.7
Total protein	7.50 (7.10, 7.90)	7.40 (7.00, 7.80)	7.51 (7.10, 8.00)	0.3
CRP	4 (2, 10)	5 (2, 14)	3 (2, 8)	0.4
PLT	150 (115, 197)	147 (108, 205)	151 (127, 182)	0.6

Metabolic parameters

Table 4 presents postoperative metabolic markers. Most values were comparable between the groups. A Wilcoxon rank sum test revealed significantly higher LDL cholesterol in the OCA group at three months (121 (102-170) vs. 107 (89-133); W=1104, p=0.027) and at six months (130 (102-156) vs. 117 (91-130); W=1083, p = 0.043).

**Table 4:** Various secondary metabolic markers *OCA: obeticholic acid; UDCA: ursodeoxycholic acid; HbA1c: hemoglobin A1c; HDL: high density lipoprotein; LDL: low density lipoprotein; VLDL: very low density lipoprotein

Characteristic	Overall, N = 83^1^	OCA, N = 41^1^	UDCA, N = 42^1^	p-value^2^
Fasting blood sugars (FBS)				
1^st^ month	119 (94, 150)	120 (97, 167)	114 (94, 138)	0.4
3^rd^ month	113 (96, 139)	112 (96, 159)	113 (96, 136)	0.7
12^th^ month	107 (95, 139)	114 (96, 147)	105 (91, 120)	0.2
HbA1C				
3^rd^ month	5.35 (4.80, 5.90)	5.30 (4.75, 5.95)	5.40 (4.80, 5.80)	>0.9
6^th^ month	5.50 (5.10, 6.00)	5.55 (5.10, 5.90)	5.50 (5.00, 6.20)	>0.9
12^th^ month	5.70 (5.20, 6.40)	5.65 (5.05, 6.50)	5.80 (5.20, 6.40)	0.8
Lipid profile: Total cholesterol				
Pre-transplant	90 (67, 120)	108 (73, 142)	81 (61, 103)	0.005
3^rd^ month	184 (158, 227)	189 (159, 239)	182 (156, 203)	0.3
6^th^ month	189 (149, 213)	195 (154, 228)	187 (147, 202)	0.3
12^th^ month	184 (151, 203)	176 (139, 207)	185 (162, 199)	0.6
HDL cholesterol				
Pre-Transplant	31 (14, 41)	31 (14, 44)	30 (18, 39)	0.4
3^rd^ month	47 (36, 60)	45 (30, 56)	51 (39, 62)	0.078
6^th^ month	47 (37, 57)	47 (34, 58)	46 (37, 56)	0.8
12^th^ month	43 (36, 51)	39 (32, 53)	44 (39, 51)	0.15
LDL cholesterol				
Pre-transplant	49 (35, 71)	53 (41, 82)	42 (33, 59)	0.014
3^rd^ month	118 (94, 146)	121 (102, 170)	107 (89, 133)	0.027
6^th^ month	121 (93, 138)	130 (102, 156)	117 (91, 130)	0.043
12^th^ month	122 (94, 144)	121 (91, 147)	122 (96, 134)	>0.9
Triglycerides				
Pre-transplant	69 (51, 99)	83 (61, 113)	59 (46, 80)	<0.001
3^rd^ month	111 (83, 152)	117 (96, 147)	93 (72, 152)	0.071
6^th ^month	106 (82, 139)	106 (82, 126)	110 (88, 160)	0.3
12^th ^month	109 (89, 131)	104 (85, 126)	119 (92, 136)	0.2
VLDL cholesterol				
Pre-transplant	13.7 (10.2, 19.7)	16.5 (11.8, 22.1)	11.6 (9.2, 15.9)	0.002
3^rd^ month	20 (16, 30)	23 (18, 27)	18 (14, 30)	0.078
6^th^ month	21 (17, 27)	20 (16, 23)	22 (18, 32)	0.13
12^th^ month	22 (17, 26)	21 (17, 25)	24 (18, 27)	0.2
^1^Median (Q1, Q3)				
^2^Wilcoxon rank sum test; Wilcoxon rank sum exact test				

Additional secondary endpoint analyses

Table 5 summarizes additional secondary endpoint analyses. No significant difference was seen in the incidence of early allograft dysfunction between groups (OCA 17% (7) vs. UDCA 12% (5); χ² = 0.001; p = 0.96). Rates of biopsy-proven rejection were similar (OCA 15% (6) vs. UDCA 12% (5), χ²=0.001; p=0.96), while treated biochemical rejection was slightly higher in the UDCA group (OCA 24% (10) vs. UDCA 36% (15); χ²=0.506; p=0.47). The incidence of biliary complications was also comparable (OCA 22% (9) vs. UDCA 31% (13), χ²=0.376; p=0.53). Pruritus was the most frequently reported adverse effect, occurring in 19.5% (n = 8) of the UDCA group and 15% (n = 6) of the OCA group (χ² = 0.335, p = 0.80). Mortality rates were lower in the OCA group than in the UDCA group (4.9% (n=2) vs. 9.5% (n=4)), although the difference was not statistically significant (χ² = 0.154, p = 0.70).

**Table 5:** Analysis of various other clinical endpoints OCA: obeticholic acid; UDCA: ursodeoxycholic acid

Characteristic	Overall, N=83^1^	OCA, N=41^1^	UDCA, N=42^1^	p-value^2^
Early allograft dysfunction	12 (14%)	7 (17%)	5 (12%)	0.96
Rejection (total)	36 (43%)	16 (39%)	20 (48%)	0.6
Biopsy-proven rejection	11 (13%)	6 (15%)	5 (12%)	0.9
Treated biochemical rejection	25 (31%)	10 (24%)	15 (36%)	0.47
Steroid-responsive rejection	24 (29%)	10 (71.4%)	14 (82.4%)	0.6
Biliary complications	22 (27%)	9 (22%)	13 (31%)	0.53
Leak	11 (13%)	7 (16.3%)	4 (9.3%)	0.3
Stricture	17 (20%)	7 (16.3%)	10 (23.3%)	0.4
Stenting	20 (24%)	9 (20.9%)	11 (25.6%)	0.7
Itching	14 (33.3%)	6 (15%)	8 (19.5%)	0.8
Mortality	6 (7.2%)	4 (9.5%)	2 (4.9%)	0.7
^1^n (%)				
^2^Pearson's Chi-squared test; Fisher's exact test				

Molecular biomarkers

Quantitative assessments of molecular markers at one month and 12 months post-transplant are presented in Table 6. A Wilcoxon rank sum test revealed significantly lower plasma bile acid levels in the OCA group (170 (108-263)) compared with the UDCA group (226 (193-317)), W=569.5; p = 0.012. All other markers, including FGF-19, TGF-β, CK-18, serum autotaxin, and BSEP, were comparable between the groups. 

**Table 6:** Quantification of molecular markers: pre-post analysis OCA: obeticholic acid; UDCA: ursodeoxycholic acid; FGF19: fibroblast growth factor 19; TGF beta: transforming growth factor beta; BSEP: bile salt export pump

Characteristic	Overall, N = 83^1^	OCA, N = 41^1^	UDCA, N = 42^1^	p-value^2^
Plasma bile acid levels post one month (ng/ml)	214 (137, 265)	170 (108, 263)	226 (193, 317)	0.012
Plasma bile acid levels: 12-month (ng/ml)	647 (578, 674)	647 (587, 670)	645 (567, 679)	>0.9
Difference in plasma bile acid levels	409 (255, 494)	469 (332, 539)	376 (229, 461)	0.006
FGF 19 post one month (pg/ml)	715 (609, 843)	751 (621, 857)	695 (588, 786)	0.3
FGF 19: 12-month (pg/ml)	162 (130, 179)	163 (130, 175)	161 (126, 187)	0.8
Difference in FGF levels	-569 (-711, -468)	-590 (-754, -466)	-555 (-642, -478)	0.4
TGF beta post one month (pg/ml)	56 (50, 94)	57 (51, 131)	54 (49, 92)	0.4
TGF beta: 12-month (pg/ml)	2,535 (1,185, 3,847)	2,699 (1,359, 3,860)	2,277 (1,003, 3,702)	0.5
Difference in TGF-beta levels	2,389 (1,135, 3,710)	2,501 (1,318, 3,814)	2,089 (952, 3,533)	0.4
Cytokeratin 18 level post one month (ng/ml)	1 (1, 5)	1 (1, 7)	1 (1, 3)	>0.9
Cytokeratin 18 level: 12-month (ng/ml)	7.3 (6.2, 9.2)	7.2 (6.0, 9.6)	7.4 (6.4, 8.3)	>0.9
Difference in cytokeratin 18 level	6 (1, 7)	5 (-1, 8)	6 (1, 7)	0.8
Serum autotaxin levels post one month (pg/ml)	138 (110, 378)	138 (95, 456)	136 (113, 344)	0.5
Serum autotaxin levels: 12-month (pg/ml)	3,168 (2,139, 4,800)	3,223 (2,361, 5,050)	3,059 (2,017, 4,562)	0.5
Difference in serum autotaxin levels	2,702 (1,704, 4,278)	2,702 (2,031, 4,062)	2,727 (1,314, 4,278)	0.5
BSEP post one month (pg/ml)	527 (252, 1,818)	422 (246, 1,173)	639 (257, 3,388)	0.4
BSEP: 12-month (pg/ml)	3,167 (1,190, 9,818)	2,417 (1,186, 9,818)	3,792 (2,233, 7,403)	0.6
Difference in BSEP levels	2,145 (265, 7,511)	1,979 (-179, 9,809)	2,448 (428, 5,549)	0.8
^1^Median (Q1, Q3)				
^2^Wilcoxon rank sum test; Wilcoxon rank sum exact test				

QoL assessment

QoL outcomes were assessed using a validated liver disease-specific questionnaire at baseline (before transplant) and at 12 months. Table 7 summarises the comparative results between the OCA and UDCA groups.

**Table 7:** Health-related quality of life assessment (n=52) OCA: obeticholic acid; UDCA: ursodeoxycholic acid

Domains	Median change in scores in OCA group (pre and post)	Median change in scores in UDCA group (pre and post)	p-value
Abdominal symptoms	-2.00 (-3, -1.7)	-2.7 (-3.17, -1.7)	0.718
Fatigue	-2 (-3, -1.2)	-2.4 (-3, -2)	0.099
Systemic symptoms	-1.6(-2.2, -0.2)	-2(-2.7, -1.4)	0.125
Activity	-1.4(-2, -0.70)	-2(-2.7, -1.4)	0.011
Emotional function	-1.65 (-2.25, -1.15)	-1.87 (-2.25, -1.47)	0.357
Worry	-1.2(-2, -0.4)	-1.4(-2.05, -1)	0.284
Total mean score	-1.43 (-2.04, -1.27)	-2.07 (-2.37, -1.44)	0.025
Total domain score	-44(-59, -34)	-57 (-66.5, -44)	0.038

The UDCA group showed a statistically significantly greater improvement in overall QoL, reflected by higher gains in both the total mean score (p=0.025) and total domain score (p=0.038). Improvements were observed across multiple domains, including well-being, fatigue, and emotional function.

## Discussion

In this randomized controlled study comparing OCA with UDCA in recipients of LDLT, the principal finding was that patients receiving OCA demonstrated a significant reduction in one-month plasma bile acid levels compared to those receiving UDCA. Under physiological conditions, plasma bile acid concentrations remain low due to efficient hepatic uptake and secretion. In cholestatic states, whether due to intrahepatic or extrahepatic causes, bile retention within hepatocytes leads to the regurgitation of bile acids into the circulation, resulting in elevated plasma levels [26]. While bile acids are essential for maintaining the bile acid pool, excessive accumulation can be hepatotoxic, underscoring the importance of tight regulation. OCA, an FXR agonist, is hypothesized to ameliorate cholestasis by enhancing hepatocellular secretion of conjugated bile acids, improving sinusoidal perfusion and clearance, and thereby reducing hepatocyte exposure to toxic bile acids [27]. Plasma bile acid concentrations decreased at one month in the OCA group, with no difference evident at the one-year assessment. This interim reduction may reflect a pharmacodynamic effect; however, longer-term data are needed to determine the clinical significance of these findings.

Both agents produced an approximate 15% reduction in cholestatic enzymes (ALP and GGT) over 12 months. However, OCA-treated patients demonstrated significantly lower ALP levels between six and 12 months and GGT levels between one and 12 months, indicating a more sustained biochemical response. These results are consistent with the POISE trial [28] and other studies [29], which showed significant improvements in ALP and bilirubin in PBC patients treated with OCA. The smaller effect size in our cohort may be due to the lower OCA dose (5 mg) used here, as prior studies employed 10-50 mg [27, 28]. Combination therapy with UDCA, as in the POISE trial, could be evaluated in future post-transplant studies. Treatment with OCA was associated with an elevation of LDL cholesterol levels at three and six months. This has been demonstrated in previous studies and is hypothesized to be due to its action on bile acid metabolism. FXR activation decreases bile acid production from cholesterol and downregulates hepatic LDL receptors, both of which lead to elevated serum levels [10]. At one year, the levels were comparable in both groups, perhaps due to statin therapy.

In terms of secondary outcomes, the incidence of rejection-related biochemical abnormalities was similar between treatment groups in this interim analysis. Neither biopsy-proven rejection rates nor biochemical rejection (defined as threefold elevations in liver enzymes after exclusion of other causes responding to pulsed steroids) differed between OCA and UDCA. Given the limitations associated with performing liver biopsies in LDLT recipients, particularly in the setting of thrombocytopenia or ascites, reliance on biochemical assessment remains common practice. Tacrolimus trough levels were comparable between groups, suggesting similar baseline immunosuppression. Prior studies, such as that by Barnes et al. [30], reported reduced rejection rates with UDCA when combined with cyclosporine-based regimens. Whether similar effects might be observed with FXR agonists or other bile acid modulators remains to be established.

Assessment of health-related QoL (HRQoL) using the CLDQ-9 demonstrated consistent improvement across all domains in both treatment groups during the first post-transplant year. Median change scores were negative, reflecting symptomatic relief and better overall well-being. While both OCA and UDCA groups showed comparable gains in abdominal, emotional, and worry domains, patients treated with UDCA demonstrated significantly greater improvement in activity (p = 0.011), total mean score (p = 0.025), and total domain score (p = 0.038), indicating superior enhancement of overall HRQoL, particularly in physical activity and global well-being. Improvements in fatigue and systemic symptoms also trended in favor of UDCA, though statistical significance was not reached. To minimize observer-related variability, we employed standardized and validated CLDQ questionnaires, administered by a transplant coordinator blinded to treatment assignment and biochemical data. Nevertheless, response bias from patients cannot be fully excluded and remains an inherent limitation of self-reported outcomes.

Biliary complications were detected in both treatment groups. While the UDCA group showed a numerically higher incidence, no detected differences were observed between groups, and no firm conclusions can be drawn from this interim analysis. Wang et al. [31] previously reported that early post-transplant UDCA administration reduced biliary sludge and casts within the first year following orthotopic LT, although long-term biliary complication rates were comparable to placebo. Evidence regarding the potential effect of OCA on biliary outcomes remains limited, and extended follow-up in the present study will be required to clarify any clinically meaningful differences.

Pruritus, a common symptom after LDLT and often associated with cholestasis, was reported in 15% of patients in the OCA group and 19.5% in the UDCA group, with no detected differences between treatments. Serum autotaxin levels, a biomarker linked to cholestatic pruritus, were comparable between groups. The overall incidence of pruritus in this study was substantially lower than previously reported in trials of OCA (up to 56%) [32], which may reflect the lower 5 mg dose used in this cohort. In some cases, pruritus persisted despite discontinuation of OCA, suggesting that the symptom was multifactorial and not necessarily attributable to the study drug.

Other secondary endpoints, including EAD, liver function parameters, and lipid and glycemic profiles, showed no detected differences between the treatment groups at this interim stage. Mortality rates were also similar, with no deaths attributed to study medications. Participants who died within one month of transplantation were excluded from analysis as per the study protocol. Importantly, as this interim analysis was not powered to detect equivalence in safety or clinical outcomes, these findings should be interpreted with caution and confirmed upon completion of the study.

Levels of FGF-19, an enterokine induced by FXR activation, showed no significant difference in the OCA group compared with the UDCA group. Other molecular markers, including CRP, TGF-β, cleaved CK-18, and serum autotaxin, also showed no detected differences between groups, consistent with the expectation that fibrosis and apoptosis are limited during the first post-transplant year.

While these interim results contribute to understanding the management of post-transplant cholestasis, ongoing vigilance is warranted regarding drug safety. In December 2024, the U.S. FDA issued a Drug Safety Communication citing an increased risk of serious liver injury, including liver failure, transplantation, and death, among non-cirrhotic patients with PBC treated with OCA [33]. Although the present study population (LDLT recipients with secondary cholestasis) differs fundamentally from that cohort, this regulatory development highlights the importance of close hepatic monitoring during OCA therapy, even in post-transplant settings.

Our results are clinically meaningful given the limited therapeutic options for managing post-transplant cholestasis. However, emerging safety concerns related to OCA use necessitate cautious interpretation. Longitudinal follow-up and comprehensive safety assessment will be critical to establishing the long-term tolerability of OCA in this unique context.

Strengths and weaknesses of our study

This study represents the first randomized controlled trial assessing the efficacy of OCA in the post-LDLT setting. The inclusion of biochemical and cellular markers of bile acid transport adds mechanistic insights into its effects on cholestasis. However, several limitations must be acknowledged. The study was open-label, and blinding was not feasible due to differences in tablet appearance and dosing frequency, though a double-dummy design could have mitigated this. Although the calculated sample size was 108 patients per group, this interim analysis included data from only about 41 patients per group who had completed follow-up at the time of assessment, which may limit statistical power and increase the risk of type II error. A final analysis will be reported once all participants complete follow-up. Furthermore, the absence of a combined OCA-UDCA treatment arm precluded assessment of potential synergistic effects.

## Conclusions

In this interim ITT analysis, both OCA and UDCA demonstrated biochemical improvement following LDLT, with comparable safety profiles. UDCA showed greater improvement in patient-reported QoL measures, while OCA produced early decrement in plasma bile acid levels. Long-term data will clarify the clinical relevance and durability of these findings.

## Figures and Tables

**Figure 2:**
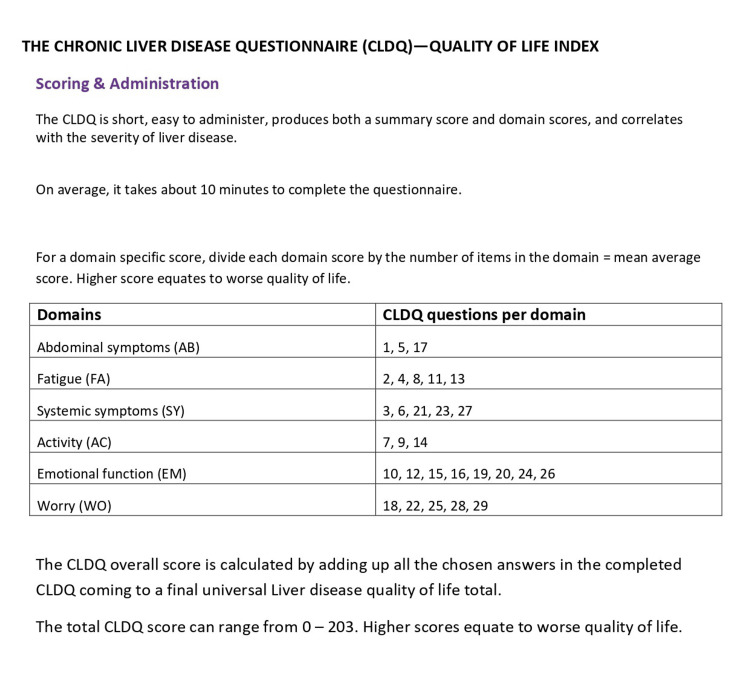
Chronic Liver Disease Questionnaire used in the study Source: [25]

**Figure 3:**
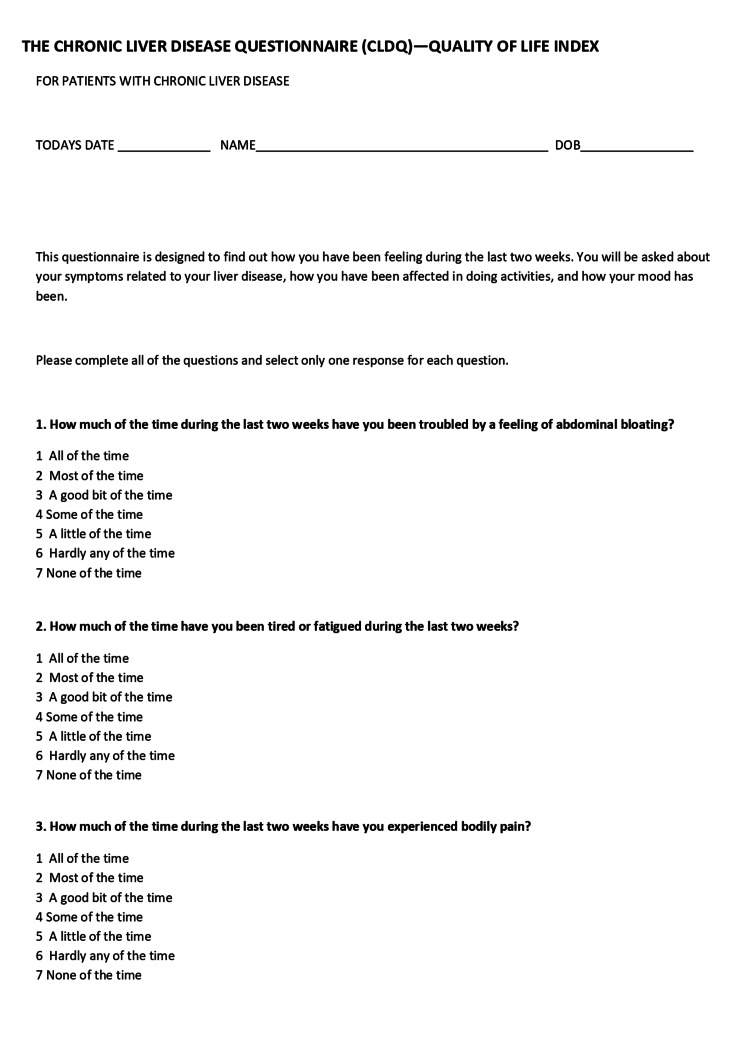
Chronic Liver Disease Questionnaire used in the study Source: [25]

**Figure 4:**
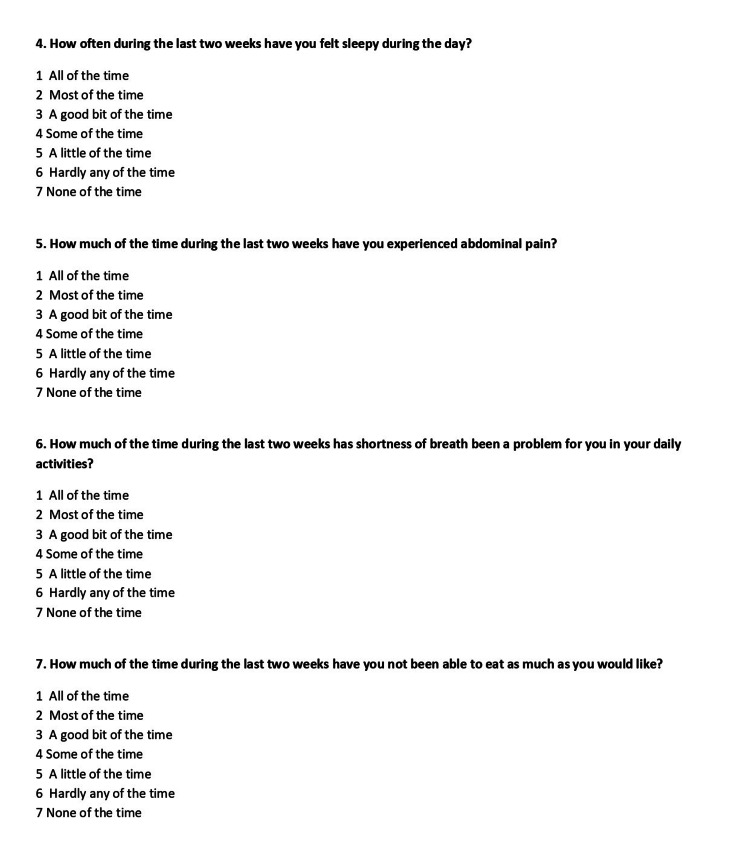
Chronic Liver Disease Questionnaire used in the study Source: [25]

**Figure 5:**
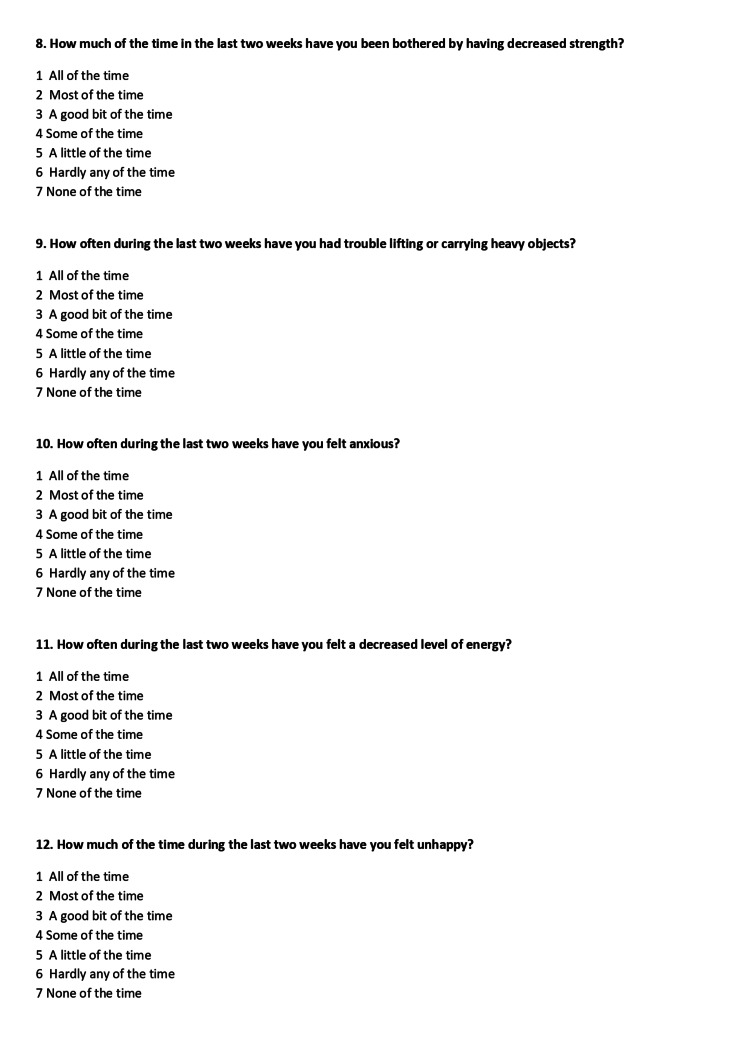
Chronic Liver Disease Questionnaire used in the study Source: [25]

**Figure 6:**
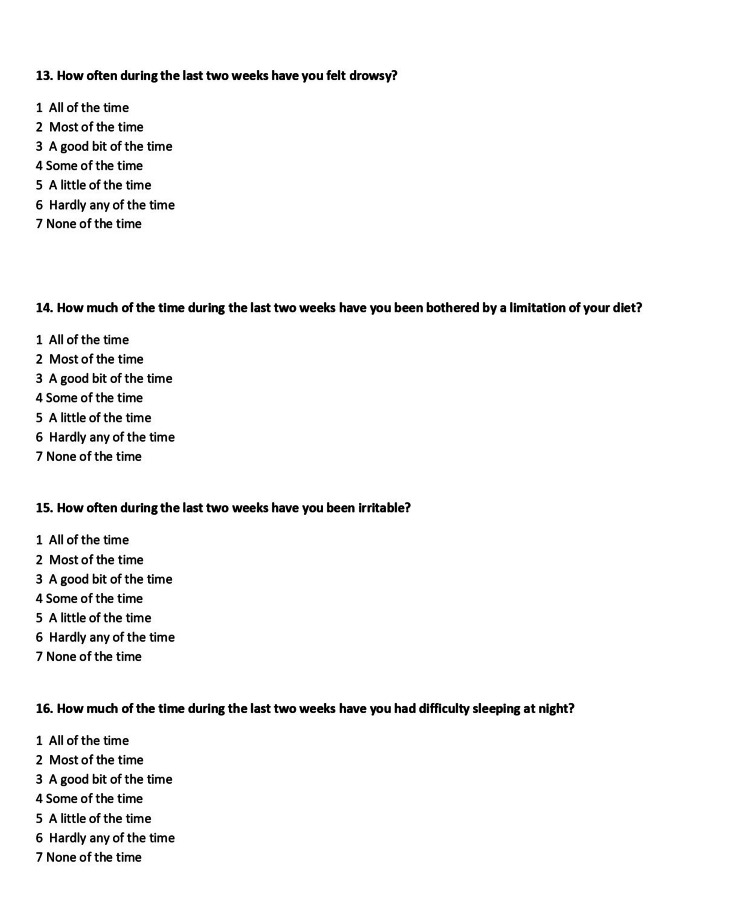
Chronic Liver Disease Questionnaire used in the study Source: [25]

**Figure 7:**
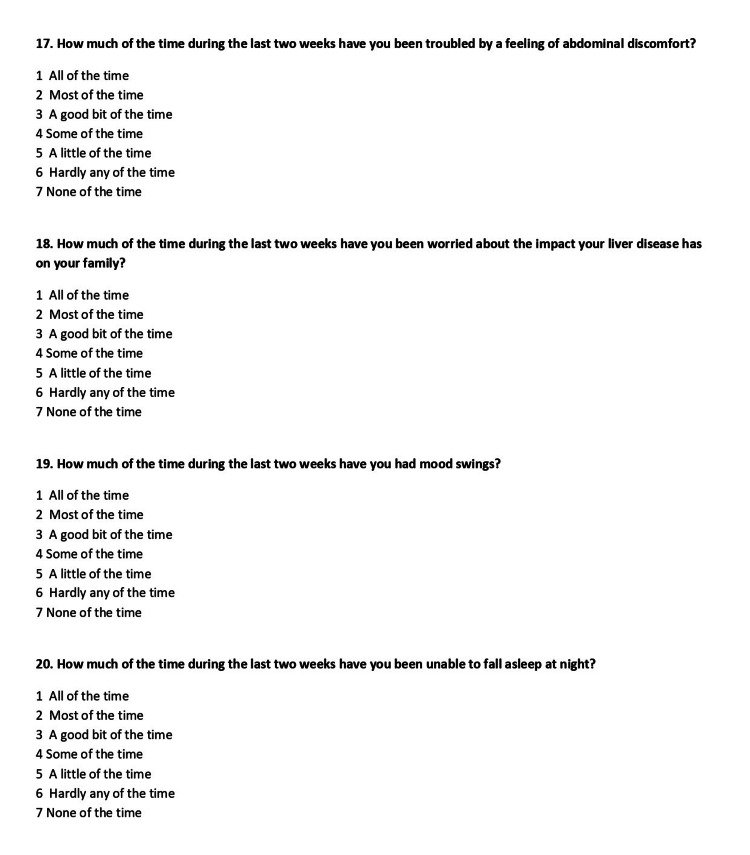
Chronic Liver Disease Questionnaire used in the study Source: [25]

**Figure 8:**
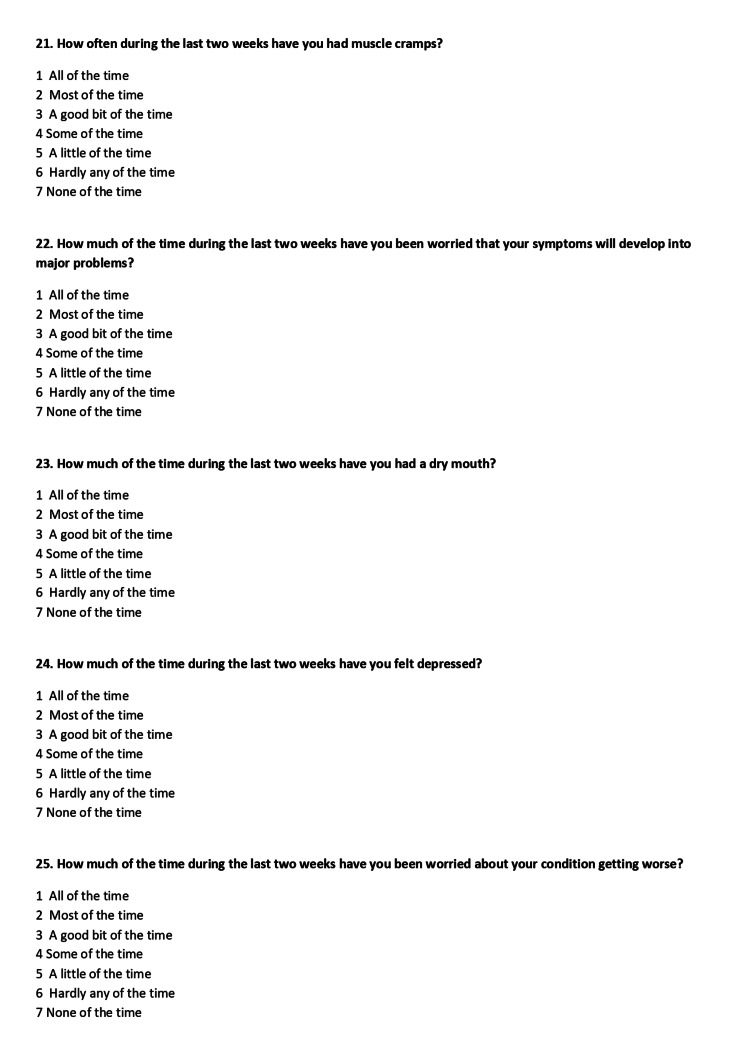
Chronic Liver Disease Questionnaire used in the study Source: [25]

**Figure 9:**
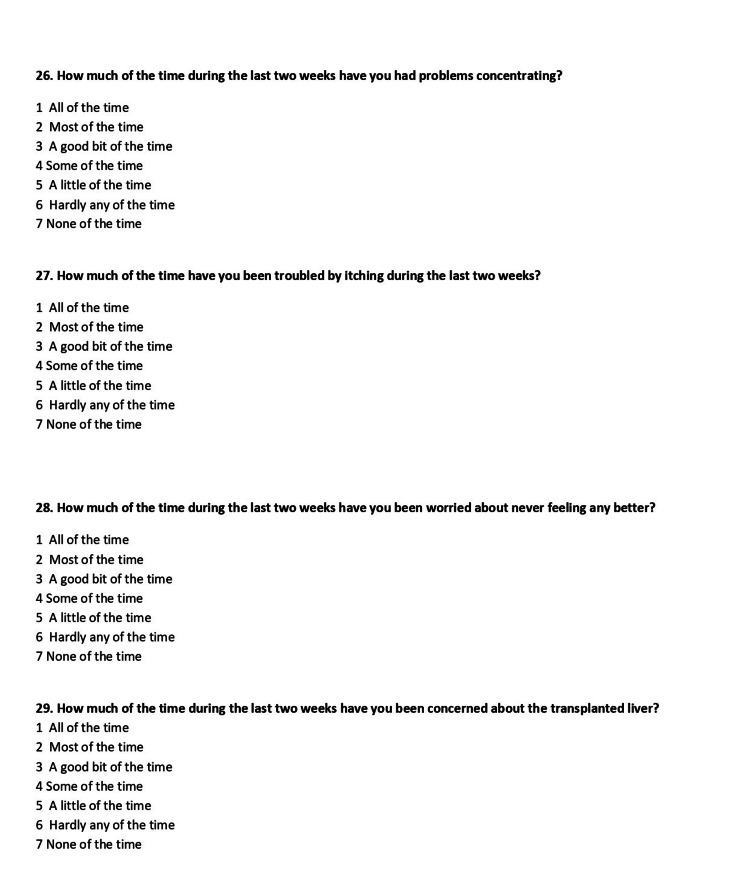
Chronic Liver Disease Questionnaire used in the study Source: [25]

## Appendices

Appendix A 
